# Genetic Comparison of Stemness of Human Umbilical Cord and Dental Pulp

**DOI:** 10.1155/2016/3453890

**Published:** 2016-03-21

**Authors:** Chung-Min Kang, Hyunok Kim, Je Seon Song, Byung-Jai Choi, Seong-Oh Kim, Han-Sung Jung, Seok-Jun Moon, Hyung-Jun Choi

**Affiliations:** ^1^Department of Pediatric Dentistry, College of Dentistry, Yonsei University, Seoul, Republic of Korea; ^2^Department of Oral Biology, Division of Histology, College of Dentistry, Yonsei University, Seoul, Republic of Korea; ^3^Department of Oral Biology, Division of Pharmacology, College of Dentistry, Yonsei University, Seoul, Republic of Korea

## Abstract

This study focuses on gene expression patterns and functions in human umbilical cord (UC) and dental pulp (DP) containing mesenchymal stem cells (MSCs). DP tissues were collected from 25 permanent premolars. UC tissue samples were obtained from three newborns. Comparative gene profiles were obtained using cDNA microarray analysis and the expression of tooth development-associated and MSC-related genes was assessed by the quantitative real-time reverse transcription polymerase chain reaction (qRT-PCR). Genes related to cell proliferation, angiogenesis, and immune responses were expressed at higher levels in UC, whereas genes related to growth factor and receptor activity and signal transduction were more highly expressed in DP. Although UC and DP tissues exhibited similar expression of surface markers for MSCs, UC showed higher expression of CD29, CD34, CD44, CD73, CD105, CD146, and CD166. qRT-PCR analysis showed that CD146, CD166, and MYC were expressed 18.3, 8.24, and 1.63 times more highly in UC, whereas the expression of CD34 was 2.15 times higher in DP. Immunohistochemical staining revealed significant differences in the expression of genes (*DSPP*,* DMP1*, and* CALB1*) related to odontogenesis and angiogenesis in DP. DP and UC tissue showed similar gene expression, with the usual MSC markers, while they clearly diverged in their differentiation capacity.

## 1. Introduction

Mesenchymal stem cells (MSCs) have attracted a great deal of interest because of their potential application in regenerative medicine and tissue engineering. MSCs are highly proliferative and adherent fibrotic cells that express characteristic cell surface markers and retain self-renewing capacity with the potential of differentiating into various tissues including bone, muscle, cartilage, fat, and nerve [1]. MSCs may be isolated readily from several tissues such as bone marrow, adipose tissue, and placenta [2]. Although MSCs derived from bone marrow are well characterized, the harvest of stem cells from the bone marrow is a highly invasive procedure with a considerable risk of donor site morbidity.

Recently, potential alternative sources of MSCs and nonaggressive methods for the harvesting of stem cells have been investigated; in particular, the umbilical cord (UC) and dental pulp (DP) show great promise as source tissues because they contain a considerable number of cells with properties similar to those of MSCs [3, 4]. In recent years, UC-MSCs have shown an odontogenic differentiation potential to differentiate into odontoblast-like cells in an odontogenic microenvironment [5].

UC tissue (Wharton's jelly of human umbilical cord) is considered an ideal source of stem cells with characteristics similar to those of MSCs from bone marrow and adipose tissue, for example, fibroblast morphology, surface protein markers, and potential for differentiation into cells of therapeutic value [6, 7]. UC cells exhibit immunosuppressive capacity and high proliferation rates, which are useful in allogeneic environments [8, 9]. DP, which is thought to be derived from migratory neural crest cells during development, is a source of various populations of multipotent stem cells [10]. Similar to MSCs, dental pulp stem cells (DPSCs) are adherent clonogenic cells with varying capacities for differentiation along mesenchymal or nonmesenchymal lineages [11].

The UC is a potentially valuable source of MSCs as the isolation process is noninvasive and causes no harm to the mother or infant and utilizes material that is usually discarded [12]. In addition, UC tissue is derived from the neonate and is therefore less mature than adult tissues. This lowers the risk of immune reactions during donor transplantation. DPSCs also represent a valuable source of MSCs, as the latter may be obtained with minimal pain and morbidity [13]. Therefore, MSCs derived from the UC and DP are more desirable for stem cell-based therapy than stem cells obtained from conventional sources such as bone marrow.

Despite extensive knowledge of the properties of UC-MSCs and DPSCs, it is still not known whether their properties accurately reflect their true gene expression patterns and developmental potential in situ. The fate of stem cells is regulated by cell-intrinsic determinants and signals within a specialized microenvironment [14]. Therefore, investigation of the genes related to stemness in UC and DP is necessary in order to evaluate the potential value of these tissues as alternative sources of MSCs. In particular, their gene expression patterns must be analyzed to obtain insights into the differentiation capacity of the stem cells and to compare the biological functions of the stemness-related genes in terms of interactions with the microenvironment. In this study, we performed a DNA microarray-based differential gene expression analysis of UC and DP tissues with the aim of comparing the characteristics of MSCs from each type of tissue.

## 2. Materials and Methods

### 2.1. Tissue Samples

The experimental protocol was approved by the Institutional Review Board of Yonsei University Dental Hospital (#2-2012-0001) and Severance Hospital (#4-2012-0408). All the subjects or their guardians have provided written informed consent. Pulp samples were obtained from healthy permanent premolars (*n* = 25; from 5 males and 6 females, aged 11–25 years) extracted for orthodontic reasons. The fresh umbilical cord tissues were obtained from three newborns in the Department of Obstetrics and Gynecology, Severance Hospital, Yonsei University. The extracted teeth and umbilical cords were frozen immediately and stored in liquid nitrogen. The pulp tissue was obtained using sterile tweezers and barbed broaches. The UC tissue was sliced at a thickness of 10–14 *μ*m using a cryostat (CM3050S, Leica Biosystems, Newcastle Upon Tyne, UK). Subsequently, the DP and UC tissues were immediately submerged in Buffer RLT, which is a proprietary component of the RNeasy Fibrous Mini Kit® (Qiagen, Valencia, CA, USA).

### 2.2. RNA Extraction

We used similar study procedures applied by Song et al. [15], Lee et al. [16], and Kim et al. [17]. Total RNA was extracted from DP and UC tissues using the RNeasy Fibrous Mini Kit (Qiagen) according to the manufacturer's instructions. The extracted RNA was eluted in 25 *μ*L of sterile water. Prior to RNA extraction, tissues were homogenized using a Bullet Blender® Bead (Next Advanced, Averill Park, NY, USA). RNA concentrations were determined from absorbance values at a wavelength of 260 nm using a spectrophotometer (Nanodrop ND-1000®, Thermo Scientific, Waltham, MA, USA). RNA samples used in this study had 260/280 nm ratios equal to or greater than 1.8.

### 2.3. cDNA Microarray

Global gene expression analyses were performed using GeneChip® Human Gene 1.0 ST oligonucleotide arrays (Affymetrix, Santa Clara, CA, USA). The average amount of RNA isolated from DP and UC tissues was 1 *μ*g. Total RNA was isolated using the RNeasy Fibrous Mini Kit columns as described by the manufacturer (Qiagen). RNA quality was assessed using the Agilent 2100 Bioanalyzer with an RNA 6000 Nano Chip® (Agilent Technologies, Amstelveen, Netherlands). RNA quantity was determined using the Nanodrop ND-1000. Each RNA sample was subjected to global gene expression analysis according to the manufacturer's protocol (http://www.affymetrix.com/). Briefly, 300 ng of total RNA from each sample was converted to double-stranded cDNA. Using a random hexamer incorporating a T7 promoter, amplified RNA (cRNA) was generated from the double-stranded cDNA template via an in vitro transcription reaction and was purified using the Affymetrix sample cleanup module. cDNA was generated by random-primer reverse transcription using a dNTP mix containing dUTP. The cDNA was then fragmented using the restriction endonucleases uracil-DNA glycosylase and human apurinic/apyrimidinic endonuclease. Next, the fragmented cDNA was end-labeled via a terminal transferase reaction incorporating a biotinylated dideoxynucleotide. Fragmented end-labeled cDNA was hybridized to the GeneChip Human Gene 1.0 ST array for 16 h at 45°C and 60 rpm as described in the GeneChip Whole Transcript Sense Target Labeling Assay Manual (Affymetrix). After hybridization, the chips were stained and washed in a GeneChip Fluidics Station 450 and scanned using a GeneChip Array scanner 3000 G7 (Affymetrix). The image data were extracted using Command Console software 1.1 (Affymetrix) and a raw file containing the expression intensity data was generated and used for the next step. This microarray data set was approved by the Gene Expression Omnibus (GEO) (https://www.ncbi.nlm.nih.gov/geo/); the GEO accession numbers of the data set are GSE75642 (umbilical cord) and GSE75644 (dental pulp).

### 2.4. Gene Ontology Analysis

Expression data were generated using Expression Console software version 1.1 (Affymetrix). For normalization, the Robust Multiarray Average algorithm of the Expression Console software was used. In order to determine whether genes were differentially expressed in the three groups, a one-way ANOVA was performed on the Robust Multiarray Average expression values. A multiple testing correction was applied to the *p* values of the *F*-statistics to adjust the false discovery rate. Genes with adjusted *F*-statistic *p* values < 0.05 were extracted. Highly expressed genes that showed over 2-fold differences between the signal values in the control and each test group were selected for further investigation. In order to classify the coexpression gene group with a similar expression pattern, we performed hierarchical and *K*-mean clustering using MultiExperiment Viewer software 4.4 (http://www.tm4.org/, Dana-Farber Cancer Institute, Boston, MA, USA). The web-based tool, DAVID (the Database for Annotation, Visualization, and Integrated Discovery), was used for biological interpretation of differentially expressed genes. Subsequently, these genes were classified based on their function according to the KEGG Pathway database (https://david.ncifcrf.gov/home.jsp).

### 2.5. Quantitative Reverse Transcription Polymerase Chain Reaction

The single-stranded cDNA required in the PCR analysis step was produced using 500 ng of extracted total RNA as a template for reverse transcription (Superscript III Reverse Transcriptase and Random Primer, Invitrogen, Paisley, UK). The reverse transcription reaction was performed at 65°C for 5 minutes, followed by 25°C for 5 minutes, 50°C for 1 hour, and 70°C for 15 minutes to inactivate the reverse transcriptase. The synthesized cDNA was diluted 10 : 1 in distilled water and used as a template for qRT-PCR, which was performed using the ABI7300 RT-PCR system (Applied Biosystems, Warrington, UK). Samples of 25 *μ*L containing 1x Universal TaqMan Master Mix (4369016, Applied Biosystems), PCR primers at a concentration of 0.9 *μ*M, and the diluted cDNA were prepared in triplicate. The amplification conditions were 50°C for 2 minutes and 95°C for 10 minutes followed by 40 cycles of 95°C for 15 seconds and 60°C for 1 minute. TaqMan gene expression assay primers (Applied Biosystems) were used. The primers for each gene are listed in Table 1. ABI 7300 SDS 1.3.1 software (Applied Biosystems) was used to record the fluorescence intensity of the reporter and quencher dyes. Fluorescence intensity values were plotted against time and quantified as the cycle number. A precise quantification of the initial target was obtained by examining the amplification plots during the early log phase of product accumulation above background (the threshold cycle (Ct) number). Ct values were subsequently used to determine ΔCt values (ΔCt = Ct of the gene minus Ct of the 18S rRNA gene control), and differences in Ct values were used to quantify the relative amount of PCR product, expressed as the relative change by applying the equation 2^−ΔCt^. The specific primer assay ID and product sizes for each gene are listed in Table 1.

### 2.6. Immunohistochemical Staining

For IHC staining, tissues from permanent teeth and the UC were fixed in 10% buffered formalin (Sigma-Aldrich, St. Louis, MO, USA) for 1 day. Permanent teeth were decalcified using 10% EDTA (pH 7.4; Thermo Fisher Scientific, Houston, TX, USA) for 8 weeks. The permanent teeth and UC tissues were embedded in paraffin and sectioned at a thickness of 3 *μ*m. Specimens were subjected to IHC staining with antibodies against DSPP (rabbit polyclonal, diluted 1 : 1500; sc-33586, Santa Cruz Biotechnology, Santa Cruz, CA, USA), DMP1 (rabbit polyclonal, diluted 1 : 100; Ab82351, Abcam), CALB1 (rabbit polyclonal, diluted 1 : 400; Ab25085, Abcam), and CD146 (MCAM, rabbit polyclonal, diluted 1 : 400; Ab75769, Abcam). Endogenous peroxidase activity was quenched by the addition of 3% hydrogen peroxide. Sections were incubated in 5% bovine serum albumin to block nonspecific binding. The primary antibodies were diluted to obtain optimal staining and sections were incubated overnight. After incubation, the EnVision+ System-HRP Labeled Polymer Anti-Rabbit kit (K4003, Dako, Carpinteria, CA, USA; ready to use) was applied for 20 minutes. Color development was performed using labeled streptavidin biotin kits (Dako) according to the manufacturer's instructions. The sections were counterstained with Gill's hematoxylin (Sigma-Aldrich). Control sections were treated in the same manner but without primary antibodies.

## 3. Results

### 3.1. Gene Expression Profiles of UC and DP Tissues

Complementary DNA microarray technology was used to compare multiple gene expression profiles representative of DP and UC tissues. In order to investigate these differentially expressed genes further, data with a more stringent threshold of 3-fold differential expression were filtered to ensure biological significance. The results indicated that 1,957 out of 33,297 (5.88%) genes exhibited an absolute expression change at least 3-fold. The expression levels of 988 genes were 3-fold higher in the UC than in DP tissues, while the expression levels of 969 genes were at least 3-fold higher in DP than in UC tissues. The data were further filtered, and the genes are listed in Tables 2 and 3 according to their biological functions.

### 3.2. Gene Ontology Analysis

**Figure 1:**
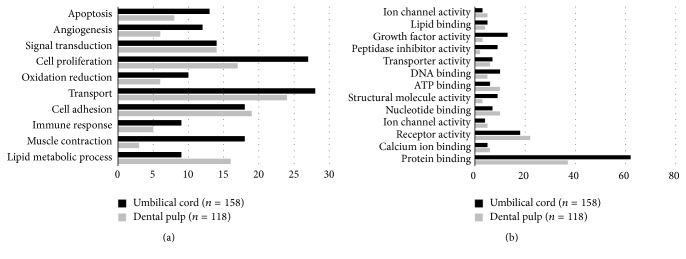
Main categories of genes expressed specifically in umbilical cord and dental pulp classified according to their biological (a) and molecular functions (b). Genes related to cell proliferation and angiogenesis were expressed at higher levels in the UC than in DP (*p* < 0.05).

In the UC tissue, the expression levels of 158 genes were upregulated 10-fold or more in comparison with DP, whereas the expression levels of 118 genes were upregulated 10-fold in DP in comparison with UC. Genes related to cell proliferation and angiogenesis were expressed at higher levels in UC than in DP. Lipid metabolic process-related genes were highly expressed in DP. In comparison with DP, a large proportion of the UC genes were related to growth factor activity, structural molecular activity, DNA binding, and protein binding (Figure 1).

### 3.3. Stemness Characterization Using Surface Protein Markers

**Figure 2:**
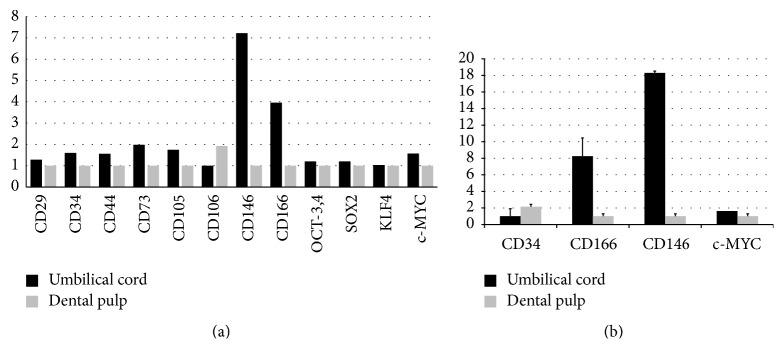
Relative gene expression of mesenchymal stem cell and induced pluripotent stem cell markers using cDNA microarray (a). The relative differences in the expression of stem cell markers between umbilical cord and dental pulp were analyzed using qRT-PCR (b). Data are presented as means ± standard deviation and expressed as the relative change by applying the equation 2^−ΔCt^, where ΔCt = Ct of the gene minus Ct of the 18S rRNA.

**Figure 3:**
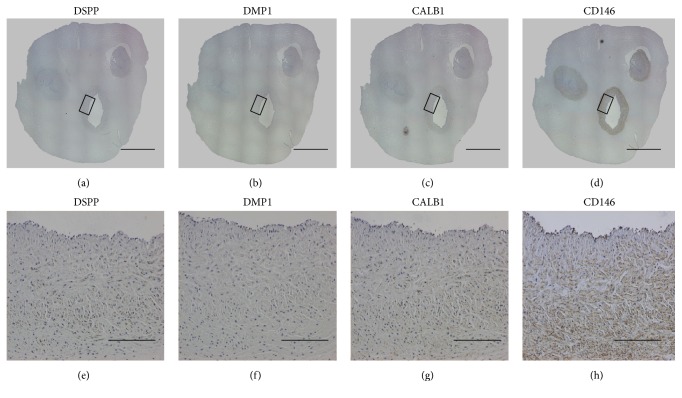
Immunohistochemical (IHC) staining of umbilical cord tissues. IHC staining for DSPP (a, e), DMP1 (b, f), CALB1 (c, g), and CD146 (d, h). CD146 is expressed on arteries of the umbilical cord, suggesting that the majority of stem cells arise from the microvasculature. (scale bars: (a)–(d) 4 mm, (e)–(h) 200 *μ*m).

The comparative expression results for MSC surface protein markers are indicated in Figure 3. UC tissue appeared to contain a population of cells that were more positive for MSC markers (including* CD29*,* CD34*,* CD44*,* CD73*,* CD105*,* CD146*, and* CD166*) according to the minimal criteria of the International Society for Cell Therapy [18]. The comparative expression analysis of four induced pluripotent stem cell (iPSC) marker genes (i.e.,* OCT4*,* SOX2*,* MYC*, and* KLF4*) revealed that the expression level of these genes was the same in UC and DP. qRT-PCR analysis for eight important marker genes revealed four tooth-related genes (*DSPP*,* AMBN*,* CALB1*, and* DMP1*), three genes for MSCs (*CD34*,* CD146*, and* CD166*), and MYC related to iPSC stemness. DSPP, AMBN, CALB1, and DMP1 were expressed in DP tissue but they were not excluded in the comparative qPCR results because they were not detected in UC tissue. The expression levels of CD146, CD166, and MYC were 18.3, 8.24, and 1.63 times higher, respectively, in UC than in DP, and the expression level of CD34 was 2.15 times higher in DP than in UC (Figure 2).

### 3.4. Immunohistochemical Staining

**Figure 4:**
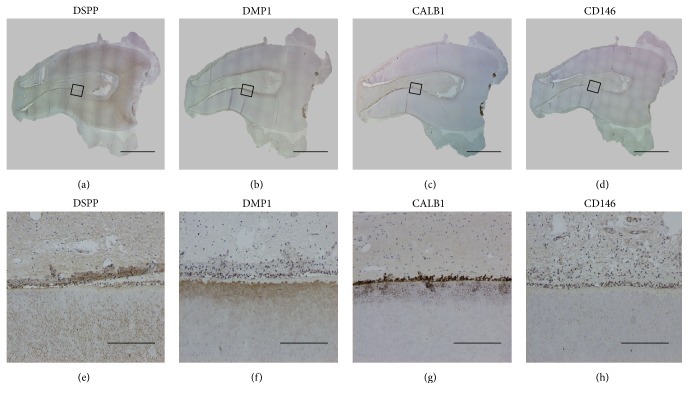
Immunohistochemical (IHC) staining of dental pulp tissue. IHC staining for DSPP (a, e). DSPP was noted in pulpal tissue, the odontoblast layer, and primary and secondary dentin. IHC staining for DMP1 (b, f) and CALB1 (c, g). CALB1 was especially expressed in pulpal tissue and the odontoblast layer. IHC staining for CD146 (d, h) (scale bars: (a)–(d) 4 mm, (e)–(h) 200 *μ*m).

MCAM (CD146) was expressed abundantly as a perivascular stem cell marker in connective tissue, especially in the arteries of the UC. However, this protein showed a very low level of expression in DP tissue (Figure 3). IHC staining showed that DSPP, DMP1, and CALB1 were expressed broadly in DP tissue (Figure 4), but barely expressed in the UC. DSPP was evident in pulpal tissue, the odontoblast layer, and primary and secondary dentin. While DMP was stained in the odontoblast layer and secondary dentin, CALB1 was expressed in pulpal tissue and the odontoblast layer. These findings were consistent with the microarray data.

## 4. Discussion

Although numerous studies have been performed to evaluate the utility of human MSCs as sources for the development of cell-based therapeutics, a precise understanding of the biology of MSCs remains elusive. Previous studies, based on a strict definition of MSCs at the molecular or cellular levels, involved serial analyses of gene expression. However, such approaches raised concerns that the expression of housekeeping genes may have interfered with the identification of MSC-specific genetic characteristics. To overcome such shortcomings, we examined DNA microarray-based differential expression profiles of MSC population, comparing these profiles with those of other tissues to evaluate stemness capacity. We compared DP with UC tissue as both types contain stromal stem cell populations with high proliferative potential that are capable of regenerating their respective microenvironments with remarkable fidelity [19]. In order to identify genes expressed at higher levels in UC than in DP tissue, we categorized genes according to the ratio of the fold change between the two tissues. Results of qRT-PCR and IHC staining analyses confirmed the validity of these data, indicating that the expression of representative genes was consistent with the differential expression patterns observed in the microarray data.

Although MSCs originating from both tissues are highly similar, their differences may be functionally related to their origin; for example, MSCs derived from DP are more committed to the osteoblastic and odontogenic lineages, whereas MSCs derived from the UC would be more committed to angiogenesis. In DP tissue, microarray results indicated that certain genes (*DMP1*,* AMBN*,* DSPP*,* DLX1*,* RUNX2*,* LEF1*,* PAX9*, and* MSX1*) related to odontogenesis and biomineral tissue development were upregulated, which was in agreement with the expected results for this biological process. In addition, genes that might be related to bone and dentin mineralization, including* PHEX*,* CALB1*,* MMP20*,* ALPL*,* LHX8*, and* WNT10A*, were upregulated. Microarray data showed that the expression of* DMP1*,* DSPP*, and* CALB* that play important roles in the development of pulp tissue was 99.2, 98.1, and 41.3 times higher, respectively, in DP than in UC. qRT-PCR results indicated that the fold differences in the expression of* DMP1*,* CALB1*, and* AMBN* were not observed in the UC. Similarly, IHC staining results showed that* DMP1*,* CALB1*, and* DSPP* were not stained in the UC but were stained around the outer area of DP. The genetic pattern analysis of permanent pulp indicated that* CALB1*, a representative gene in DP, is necessary for enamel mineralization in transition- and maturation-stage ameloblasts [17].

MSCs possess multilineage differentiation potential with a variety of chemokines, cytokines, and growth factors involved in the regeneration of damaged tissue. They are capable of modifying their molecular activities and functions in response to the environment. The exclusive expression of the chemokines CXCL1 and CXCL6 in the UC may increase propagation of hematopoietic precursors in coculture settings. Other genes expressed at higher levels in the UC include those encoding IL-6, IL-18, FGF9, FGF10, PDGFA, EGF, and VEGFA, which are part of interconnected pathways related to angiogenesis. Jin et al. reported that MSCs derived from bone marrow, adipose tissue, and the UC have significantly different anti-inflammatory capacities and confirmed that UC-MSCs exhibit the greatest anti-inflammatory effects [20]. These findings suggest that UC-MSCs are more efficient for clinical applications involving revascularization.

In this study, the comparison of stemness of UC and DP tissues revealed no significant fold difference in the expression of several surface markers (CD29, CD34, CD44, CD73, CD105, and CD106) typical for MSCs. Nevertheless, some differences were observed in the expression level of CD146 (MCAM) and CD166 (ALCAM), which connect the control of cell growth with cell migration. These findings are representative of the developmental process. The qRT-PCR results showed that the expression levels of CD146 and CD166 were higher in UC than in DP (18.3-fold and 8.24-fold, resp.). These molecular differences in tissue-specific MSC gene expression may reflect their functional activities in distinct niches. A study utilizing flow cytometry reported higher expression of CD146, a marker expressed on both BMSCs and DP-MSCs [21]. IHC data confirmed that CD146 is a marker of vascular endothelial cells expressed on arteries of the UC and the outer walls of blood vessels in DP, suggesting that the majority of stem cells arise from the microvasculature. Accumulating evidence suggests that the expression of CD166 reflects the onset of a cellular program involving neural development, branching organ development, hematopoiesis, the immune response, and tumor progression [22]. Struys et al. reported that cultured DPSCs and UC-MSCs showed a similar expression pattern of antigens characteristic of MSCs such as CD105, CD29, CD44, CD146, and STRO-1 [23]. DPSCs are also identified by their positive expression of CD29, CD44, CD73, CD90, CD105, and STRO-1 [19]. CD34 protein is a specific antigen in hematopoietic cells, indicating that a greater number of immature hematopoietic cells are present in both UC and DP [24]. CD34 is present on the outer cell walls of DP and in the connective tissue of the UC, in agreement with previous studies reporting that CD34 localizes on large blood vessels, but not capillaries [25].

The expression of pluripotent stem cell genes in the UC and DP might reflect their embryonic origin. iPSCs are the most promising cell source for cell-based therapy in regenerative medicine, as they give rise to development by introducing 4 factors: MYC, KLF4, OCT4, and SOX2 [7]. No significant differences were found between the expressions of these factors in the two tissue types; MYC, KLF4, OCT4, and SOX2 were expressed 1.57, 1.03, 1.20, and 1.20 times more highly, respectively, in UC than in DP tissue. Previously, DPSCs were characterized by the low levels of expression of undifferentiated cell-associated genes, such as OCT4, MYC, and Nanog, which are considered to facilitate reprogramming [26]. Recent studies utilizing the UC to derive iPSCs are expected to contribute to the further expansion of its pluripotency for therapeutic purposes, including drug discovery [27, 28].

The UC expresses specific embryonic cell markers such as DLK1, DKK1, TBX18, WNT4, and TGFB3. Previous studies describing the expression of embryonic cell markers in the UC have shown that fetal perivascular cells express Runx1 and OCT-4 at different levels, which characterizes the undifferentiated stem cell state [29]. Interestingly, UC-MSCs exhibited higher levels of expression of genes related to cell proliferation than DPSCs, whereas DPSCs exhibited a higher proliferation rate compared with BMSCs in vitro [11]. Previous study revealed similar result of this research that UC-MSCs seemed to have higher cell proliferation ability, while DP-MSCs may have significant differences for lower cell apoptosis, osteogenic differentiation, and senescence [30]. This may be attributed to the developmental state of tissues, as UC samples are at an earlier stage of development compared with DP from fully developed and erupted permanent teeth. Commonly expressed genes in DP include those coding for various growth factors (BMP-2, BMP-5, BMP-7, MMP20, and TGF-*β*1) implicated as strong promoters of the formation of mineralized bone matrix and tooth morphogenesis [31]. Coexpression of genes with known functions, and unknown or novel genes, may provide a simple means to obtain data about genes for which little information is available.

Although the results of this study are still limited and require further investigation using additional methods, the similarity between MSCs derived from the UC and DP at the transcription level definitively places both tissues as potentially more accessible sources of MSCs. Furthermore, the present gene expression analysis confirms similarities between MSCs derived from the UC and DP and provides molecular and biological insights into the developmental mechanisms involved in angiogenic and odontogenic processes.

## 5. Conclusions

Here, we presented comparative gene expression data for human UC and DP tissue. Although UC tissue showed similar but slightly higher expression patterns, with the usual MSC markers, both tissues clearly diverged in their differentiation capacity. Further research is necessary to understand and describe the significance of these findings for clinical applications.

## Figures and Tables

**Table 1:** Quantitative RT-PCR primer lists.

Gene symbol	Gene description	Assay ID	Amplicon length
AMBN	Ameloblastin (enamel matrix protein)	Hs00212970_m1	61
ALCAM (CD166)	Activated leukocyte cell adhesion molecule	Hs00977641_m1	103
CALB1	Calbindin 1	Hs00191821_m1	90
CD34	CD34 molecule	Hs00990732_m1	91
DMP1	Dentin matrix acidic phosphoprotein 1	Hs01009391_g1	106
DSPP	Dentin sialophosphoprotein	Hs00171962_m1	67
MCAM (CD146)	Melanoma cell adhesion molecule	Hs00174838_m1	77
MYC	c-Myc	Hs00153408_m1	107
18S	18S rRNA	Hs03003631_g1	69

**Table 2:** Representative differentially expressed genes with higher expression levels in umbilical cord than in dental pulp tissue.

Functional category	Gene symbol	Biological process	Accession number	Absolute fold change
Embryonic development	DLK1	Embryonic skeletal system development	NM_003836	56.86
	DKK1	Embryonic limb morphogenesis	NM_012242	44.51
	SCEL	Epidermis development, embryo development	NM_144777	33.26
	TBX18	Morphogenesis of embryonic epithelium	NM_001080508	15.66
	HOXD10	Embryonic skeletal system morphogenesis	NM_002148	12.03
	TBX20	Embryonic heart tube development	NM_001077653	11.71
	HOXC10	Embryonic limb morphogenesis	NM_017409	8.70
	HOXC6	Embryonic skeletal system development	NM_004503	7.22
	HAND2	In utero embryonic development	NM_021973	6.54
	HOXA6	Embryonic skeletal system morphogenesis	NM_024014	5.50
	EDN1	In utero embryonic development	NM_001955	5.35
	WNT4	Embryonic epithelial tube formation	NM_030761	4.31
	GATA6	In utero embryonic development	NM_005257	4.17
	TGFB1I1	Morphogenesis of embryonic epithelium	NM_001042454	4.13
	TGFB3	In utero embryonic development	NM_003239	3.85
	SOX5	In utero embryonic development	NM_152989	3.70

Developmental process	DES	Cytoskeleton organization	NM_001927	66.18
	KRT6A	Ectoderm development	NM_005554	27.69
	KRT5	Epidermis development	NM_000424	22.73
	KRT13	Epidermis development, tongue morphogenesis	NM_153490	22.53
	COL12A1	Skeletal system development, collagen fibril organization	NM_004370	17.87
	DKK2	Multicellular organismal development	NM_014421	12.63
	TAGLN	Muscle organ development	NM_001001522	11.91
	KRT8	Cytoskeleton organization	NM_002273	11.04
	KRT14	Epidermis development	NM_000526	9.01
	OSTN	Ossification, multicellular organismal development	NM_198184	8.22
	MCAM	Anatomical structure morphogenesis	NM_006500	7.22
	IGF2BP3	Anatomical structure morphogenesis	NM_006547	7.10
	ADAM19	Fertilization, muscle development, neurogenesis	NM_033274	6.41
	MGP	Cartilage condensation, ossification	NM_001190839	5.70
	DMD	Muscle organ development, skeletal muscle tissue development	NM_000109	5.22
	PITX2	Multicellular organismal development	NM_153426	4.79

Physiological process	ACTG2	Muscle contraction	NM_001615	115.24
	HBG1	Transport	NM_000559	101.65
	PLN	Cellular calcium ion homeostasis, blood circulation	NM_002667	71.69
	CNN1	Regulation of smooth muscle contraction	NM_001299	66.37
	HBB	Regulation of blood pressure, oxygen transport	NM_000518	38.48
	FGF10	Angiogenesis	NM_004465	31.24
	COL8A1	Angiogenesis	NM_001850	12.02
	AHSP	Hemoglobin metabolic process, hematopoiesis	NM_016633	7.18
	FGF9	Angiogenesis, osteoblast differentiation	NM_002010	5.07
	PDGFA	Angiogenesis, response to hypoxia	NM_002607	5.00
	ELN	Respiratory gaseous exchange, blood circulation	NM_000501	4.75
	EGF	Angiogenesis, positive regulation of cell proliferation	NM_001963	4.30
	IL18	Angiogenesis, response to hypoxia	NM_001562	3.85
	VEGFA	Angiogenesis, ovarian follicle development	NM_001025366	3.16

Signal transduction and regulation	PRLR	Cell surface receptor linked signaling pathway	NM_000949	90.61
	RASSF3	Signal transduction	NM_178169	12.27
	RERG	GTPase mediated signal transduction	NM_032918	11.98
	STC2	Cell surface receptor linked signaling pathway	NM_003714	11.37
	CD244	Signal transduction	NM_016382	8.83
	ASPN	Negative regulation of transforming growth factor beta receptor signaling pathway	NM_017680	8.20
	LPHN3	G-protein coupled receptor protein signaling pathway	NM_015236	7.72
	SPSB1	Intracellular signaling pathway	NM_025106	6.87
	ALCAM	Signal transduction, motor axon guidance	NM_001627	3.96

Cell regulation and proliferation	UPK1B	Epithelial cell differentiation	NM_006952	45.66
	MYOCD	Cardiac cell differentiation	NM_001146312	40.32
	EGFL6	Cell differentiation	NM_015507	11.94
	PODN	Negative regulation of cell proliferation	NM_153703	9.96
	FAS	Positive regulation of necrotic cell death	NM_000043	9.34
	KRT4	Epithelial cell differentiation, negative regulation of epithelial cell proliferation	NM_002272	6.52
	IGFBP7	Regulation of cell growth	NM_001553	6.01
	HEMGN	Cell differentiation, regulation of osteoblast differentiation	NM_018437	5.70
	CDH1	Trophectodermal cell differentiation	NM_004360	5.44
	DPT	Cell adhesion, negative regulation of cell proliferation	NM_001937	5.13

Cytokine, chemokine, and immune response	IL1RL1	Immune response	NM_016232	36.22
	S100A8	Chemotaxis, inflammatory response	NM_002964	26.48
	DPP4	Regulation of T cell mediated immunity	NM_001935	18.16
	ITGB6	Inflammatory response	NM_000888	15.01
	IGF2BP1	Regulation of cytokine biosynthetic process	NM_006546	9.59
	LY96	Inflammatory response, cellular defense response	NM_015364	8.40
	CCRL1	Chemotaxis, immune response	NM_178445	7.81
	ANLN	Cytokinesis	NM_018685	7.60
	CMKLR1	Chemotaxis, immune response	NM_001142343	7.41
	CD97	Inflammatory response, immune response	NM_078481	6.96
	CXCL1	Chemotaxis, inflammatory response, immune response	NM_001511	5.54
	IL33	Positive regulation of macrophage activation	NM_033439	5.29
	CXCR1	Chemotaxis, inflammatory response	NM_000634	3.48
	CXCL6	Chemotaxis, inflammatory response	NM_002993	3.07

**Table 3:** Representative differentially expressed genes with higher expression levels in dental pulp tissue than in umbilical cord.

Functional category	Gene symbol	Biological process	Accession number	Absolute fold change
Biomineral tissue development	PHEX	Bone and dentin mineralization	NM_000444	160.01
	DMP1	Bone and dentin mineralization	NM_004407	99.20
	CALB1	Hydroxyapatite formation	NM_004929	98.07
	MMP20	Regulation of enamel mineralization, proteolysis	NM_004771	85.26
	AMBN	Bone mineralization, odontogenesis of dentine-containing tooth	NM_016519	65.69
	DSPP	Odontogenesis of dentine-containing tooth	NM_014208	41.26
	ALPL	Biomineral tissue development	NM_000478	14.83
	DLX1	Odontogenesis of dentine-containing tooth	NM_178120	9.55
	LHX8	Odontogenesis of dentine-containing tooth	NM_001001933	7.56
	CA2	Odontogenesis of dentine-containing tooth	NM_000067	7.07
	RUNX2	Ossification, osteoblast differentiation	NM_001024630	6.53
	LEF1	Odontogenesis of dentine-containing tooth	NM_016269	6.24
	PAX9	Tooth development	NM_006194	5.79
	MSX1	Odontogenesis, craniofacial development	NM_002448	4.04
	WNT10A	Regulation of odontogenesis of dentine-containing tooth	NM_025216	3.87

Developmental process	DLX5	Osteoblast differentiation	NM_005221	30.01
	DLX3	Multicellular organismal development	NM_005220	26.13
	ADAM22	Proteolysis, central nervous system development	NM_021723	25.00
	NES	Central nervous system development	NM_006617	15.67
	BMP7	Ossification, organ morphogenesis	NM_001719	15.60
	BMPR1B	Skeletal system development, cartilage condensation	NM_001203	13.53
	MSX2	Skeletal system development, osteoblast differentiation	NM_002449	9.32
	COL11A2	Skeletal system morphogenesis, cartilage development, palate development	NM_001163771	8.55
	BMP5	Skeletal system development, ossification	NM_021073	6.08
	TBX3	Blood vessel development, in utero embryonic development	NM_016569	5.93
	MBP	Central nervous system development, myelination	NM_001025101	4.95
	DLX6	Multicellular organismal development, nervous system development	NM_005222	4.12
	NRCAM	Axonogenesis, central nervous system development	NM_001193582	4.09
	SNAI1	Osteoblast differentiation, mesoderm formation	NM_005985	3.78
	HLF	Multicellular organismal development, rhythmic process	NM_002126	3.23
	BMP2	Development of bone and cartilage	NM_001200	2.11

Physiological process	TF	Controlling iron concentrations, erythropoiesis	NM_001063	153.98
	SCN7A	Sodium ion transport, muscle contraction	NM_002976	82.36
	APOD	Lipid metabolic process	NM_001647	41.22
	PLAT	Response to hypoxia, blood coagulation	NM_000930	10.20
	CD52	Elevation of cytosolic calcium ion concentration	NM_001803	4.29
	CYGB	Response to oxidative stress, oxygen transport	NM_134268	3.98
	MYOT	Muscle contraction	NM_006790	3.26

Signal transduction and regulation	WIF1	Wnt receptor signaling pathway	NM_007191	70.19
	PTN	Transmembrane receptor protein signaling pathway	NM_002825	38.17
	WNT5A	Oncogenesis, embryogenesis	NM_003392	13.68
	NCAM1	Neurite outgrowth, synaptic plasticity	NM_181351	7.87
	NCAM2	Neurite outgrowth, synaptic plasticity	NM_004540	7.38
	RASGRF2	Induction of apoptosis by extracellular signals	NM_006909	6.88
	CHN1	Signal transduction	NM_001822	6.56
	MET	Cell surface receptor linked signaling pathway	NM_001127500	3.01

Cell regulation and proliferation	SCIN	Negative regulation of cell proliferation	NM_001112706	58.65
	RELN	Cell morphogenesis involved in differentiation	NM_005045	49.12
	NES	Structural organization of the cell	NM_006617	15.67
	MEGF10	Phagocytosis	NM_032446	9.89
	EPCAM	Positive regulation of cell proliferation	NM_002354	8.79
	PDGFD	Positive regulation of cell division	NM_025208	5.43
	CLU	Cell death, positive regulation of cell proliferation	NM_001831	5.19
	VEGFC	Angiogenesis, positive regulation of neuroblast proliferation	NM_005429	3.74
	MSI2	Stem cell development	NM_138962	3.55
	DBC1	Cell cycle arrest, cell death	NM_014618	3.32
	TGFB1	Cell growth, cell proliferation, cell differentiation and apoptosis	NM_000660	3.14
	IGFBP6	Regulation of cell growth	NM_002178	3.07

Cytokine, chemokine, and immune response	IGJ	Immune response	NM_144646	22.65
	IGHD	Immune response	BC021276	11.30
	CXCL14	Chemotaxis, immune response, inhibiting angiogenesis	NM_004887	8.74
	SELE	Leukocyte migration involved in inflammatory response	NM_000450	5.82
	MX1	Induction of apoptosis, defense response, response to virus	NM_002462	5.24
	IFI44	Response to virus	NM_006417	5.05
	CX3CL1	Chemotaxis, defense response, immune response	NM_002996	3.60
	CFI	Innate immune response	NM_000204	3.17
